# Assessing the Accuracy of ChatGPT in Answering Questions About Prolonged Disorders of Consciousness

**DOI:** 10.3390/brainsci15040392

**Published:** 2025-04-13

**Authors:** Sergio Bagnato, Cristina Boccagni, Jacopo Bonavita

**Affiliations:** Villa Rosa Rehabilitation Hospital, Provincial Agency for Health Services (APSS) of Trento, 38057 Pergine Valsugana, Italy; cristina.boccagni@apss.tn.it (C.B.); jacopo.bonavita@apss.tn.it (J.B.)

**Keywords:** vegetative state, unresponsive wakefulness syndrome, minimally conscious state, large language models, chatbot, ChatGPT o1, AI in healthcare, empathy, language comparison, caregiver support

## Abstract

**Objectives**: Prolonged disorders of consciousness (DoC) present complex diagnostic and therapeutic challenges. This study aimed to evaluate the accuracy of two ChatGPT models (ChatGPT 4o and ChatGPT o1) in answering questions about prolonged DoC, framed as if they were posed by a patient’s relative. Secondary objectives included comparing performance across languages (English vs. Italian) and assessing whether responses conveyed an empathetic tone. **Methods**: Fifty-seven open-ended questions reflecting common caregiver concerns were generated in both English and Italian, each categorized into one of three domains: clinical data, instrumental diagnostics, or therapy. Each question contained a background context followed by a specific query and was submitted once to both models. Two reviewers evaluated the responses on a four-point scale, ranging from “incorrect and potentially misleading” to “correct and complete”. Discrepancies were resolved by a third reviewer. Accuracy, language differences, empathy, and recommendation to consult a healthcare professional were analyzed using absolute frequencies, percentages, the Mann–Whitney U test, and Chi-squared tests. **Results**: A total of 228 responses were analyzed. Both models provided predominantly correct answers (80.7–96.8%), with English responses achieving higher accuracy only for ChatGPT 4o on clinical data. ChatGPT 4o exhibited greater empathy in its responses, whereas ChatGPT o1 more frequently recommended consulting a healthcare professional in Italian. **Conclusions**: Both ChatGPT models demonstrated high accuracy in addressing prolonged DoC queries, highlighting their potential usefulness for caregiver support. However, occasional inaccuracies emphasize the importance of verifying chatbot-generated information with professional medical advice.

## 1. Introduction

Prolonged disorders of consciousness (DoC) are defined by impaired consciousness persisting for at least 28 days following severe brain injury [1]. While such clinical conditions may also occur in the advanced stages of neurodegenerative diseases, the term “prolonged DoC” typically refers to the sequelae of acute brain injury, such as traumatic brain injury, cerebral hypoxia, or stroke. These disorders invariably follow an initial coma phase and encompass two main diagnostic categories: unresponsive wakefulness syndrome (UWS) and minimally conscious state (MCS). UWS is characterized by spontaneous eye opening without evidence of self-awareness or environmental awareness [2], whereas MCS involves minimal and inconsistent signs of awareness [3].

Prolonged DoC are devastating not only because of their severe impact on patients, but also due to their profound emotional and social consequences for families [4,5]. These conditions place a significant psychological burden on caregivers, as their onset is sudden and unexpected—an effect further amplified by the complexity and uncertainty surrounding prolonged DoC. Even experienced clinicians often face considerable challenges in accurately distinguishing UWS from MCS, further complicating diagnostic certainty [6,7]. Prognostication remains difficult, both in terms of recovery of consciousness and long-term functional outcomes [8]. Additionally, prolonged DoC are frequently associated with complications, such as hydrocephalus, seizures, spasticity, infections, paroxysmal sympathetic hyperactivity, and critical illness neuromyopathy, all of which can further hinder recovery [9,10,11]. As a result, these patients typically require extensive care and rehabilitative treatment for months or even years, making precise and transparent communication between clinicians and patients’ families essential, ensuring that information is aligned with current scientific evidence and best clinical practices.

Since 2022, the introduction of chatbots capable of simulating human-like linguistic interaction transformed access to information on a variety of topics, including medical issues. On 30 November of that year, OpenAI, a private company, released ChatGPT [12], a chatbot that enables users to interact conversationally, reaching an unprecedented level of accuracy. In 2024, OpenAI released several new versions of its chatbot, most notably GPT-4o (where “o” stands for “omni”, meaning “all”) and its most advanced model, OpenAI o1, which, according to OpenAI, significantly outperforms GPT-4o on challenging reasoning benchmarks. OpenAI o1 was specifically developed to enhance performances on complex reasoning tasks, utilizing a method known as “chain-of-thought reasoning” [13]. This approach enables the model to work through problems step-by-step, thereby reducing hallucinations, that is, instances where the model generates plausible-sounding but incorrect or unsupported information. As a result, it may provide more accurate responses to complex medical questions compared to earlier versions of ChatGPT [14]. Consistent with the naming conventions used on the official ChatGPT app, this article refers to these models as ChatGPT 4o and ChatGPT o1, respectively. From the outset, the potential applications of ChatGPT in the medical field have generated considerable interest and debate within the scientific community. As a tool virtually accessible to any practitioner, it presents both opportunities and risks [15,16,17]. Interestingly, several studies have evaluated ChatGPT’s ability to answer medical questions across various clinical domains, reporting responses that range from accurate to potentially misleading [18,19,20,21,22,23]. Another key consideration is that the accuracy of ChatGPT may vary depending on the language used. Although it has been trained on large multilingual datasets, the majority of its training data is in English. Consequently, its performance on medical topics may differ across languages, an issue that has been rarely investigated [21].

The primary aim of this study was to evaluate the accuracy of two ChatGPT models (ChatGPT 4o and ChatGPT o1) in responding to questions about prolonged DoC, framed as if they were posed by a patient’s relative. As a secondary objective, we compared their performance in English versus Italian and assessed whether the responses conveyed an empathetic tone. The results of this study will help determine whether ChatGPT can serve as a valuable supportive tool for the families of patients with prolonged DoC.

## 2. Materials and Methods

### 2.1. Data Collection and Evaluation

This study was conducted between January and March 2025. First, a questionnaire comprising 57 open-ended questions—based on those commonly asked by family members of patients with prolonged DoC—was developed. The questions were derived from the experience of two authors (SB and CB), each with approximately 20 years of clinical practice and research experience in prolonged DoC. The questions were categorized into three domains: clinical data (*n* = 29), instrumental diagnostics (*n* = 14), and therapy (*n* = 14). Each question contained an introductory contextualization (e.g., “My relative has a prolonged disorder of consciousness due to a severe brain injury”) followed by a specific query (e.g., “Is CT useful for determining if they are conscious?”). The questionnaire was developed in both English and Italian. All 57 questions were submitted once to both ChatGPT 4o and ChatGPT o1. At the time of this study, ChatGPT-4o was freely available, albeit with daily usage limitations, while ChatGPT o1 required a paid subscription. Each question was entered as an independent prompt using the “New chat” function. Responses were graded using a four-point scale based on their accuracy, alignment with scientific evidence [24,25], and consistency with best clinical practices. The following grading system was adapted from previous studies evaluating ChatGPT’s performance [20].

1. Incorrect and potentially misleading information: the information provided is not consistent with the most recent scientific evidence or best clinical practice and could lead to conclusions unsupported by the data.
2. Partially correct information, with significant errors: the information provided contains both correct elements and elements that are not in line with the most recent scientific evidence or best clinical practice.
3. Correct but incomplete information: the information provided is consistent with the most recent scientific evidence and best clinical practice, but lacks some necessary elements to fully answer the question.
4. Correct and complete information: The information provided is fully consistent with the most recent scientific evidence and best clinical practice.

Two authors (SB and CB) independently graded each response, with discrepancies resolved by a third expert reviewer (JB).

Additionally, responses were assessed for their empathetic tone based on the explicit presence of phrases that acknowledged the sensitive emotional context of prolonged DoC (e.g., “I’m sorry to hear about your relative’s condition”). Responses were also evaluated for the inclusion of a recommendation to consult a healthcare professional for a more in-depth evaluation of the issue. Because this information was easily identifiable in the responses, it was not subjected to independent double review.

Since this study does not involve clinical data or sensitive information, ethical committee approval was not required.

### 2.2. Data Analysis

Absolute frequencies and percentages for each grade were calculated separately for responses provided by ChatGPT 4o and ChatGPT o1 in both English and Italian. Interrater reliability between the two primary reviewers was assessed using weighted Cohen’s Kappa. Comparisons between models and languages were performed using Mann–Whitney U and Chi-squared (χ^2^) tests. *p* values < 0.05 were considered statistically significant. All statistical analyses were performed using Prism 10 (GraphPad Software, San Diego, CA, USA), while figures were created in Python 3 (Python Software Foundation, Wilmington, DE, USA) using code generated by ChatGPT o1.

## 3. Results

**Figure 1:**
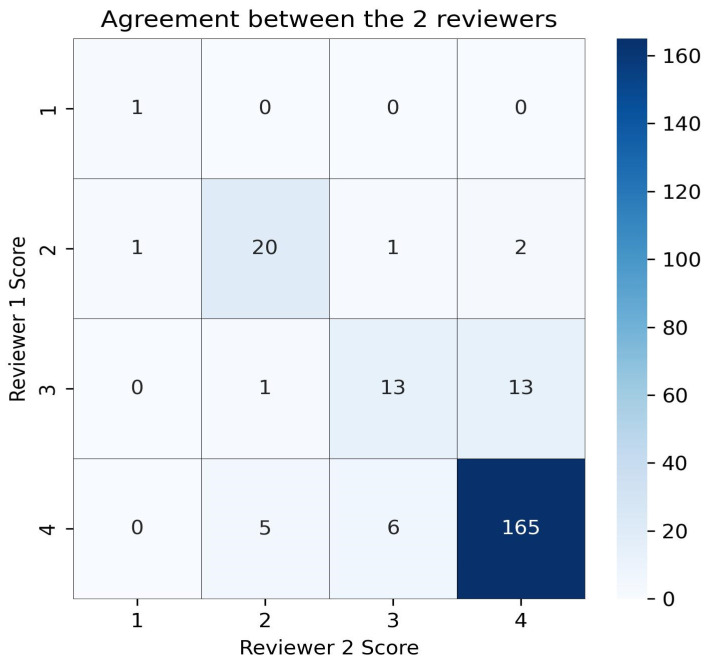
Heatmap illustrating interrater agreement between the two primary reviewers. Cell values indicate the number of responses assigned to each grading category (1 = incorrect, 4 = correct and complete) by Reviewer 1 (rows) and Reviewer 2 (columns). Darker shades correspond to higher frequencies.

A total of 228 responses were analyzed. Interrater reliability, assessed using weighted Cohen’s Kappa, was 0.76, denoting substantial agreement between the two reviewers [26] (Figure 1). Discrepancies in the 29 responses (12.7% of the total) for which initial evaluations differed were resolved by the third reviewer. All evaluated responses, along with the corresponding reviewers’ scores, are listed in the Supplementary Materials.

Of the 57 questions posed in English, ChatGPT 4o responses were graded as incorrect in 1 case (1.7%), partially correct in 3 cases (5.3%), correct but incomplete in 8 cases (14%), and correct and complete in 45 cases (78.9%). ChatGPT o1 responses were graded as incorrect in 0 cases, partially correct in 2 cases (3.5%), correct but incomplete in 8 cases (14%), and correct and complete in 47 cases (82.5%) (Table 1). Differences between the two models were not significant (U = 1559; *p* = 0.6).

Of the 57 questions posed in Italian, ChatGPT 4o responses were graded as incorrect in 1 case (1.7%), partially correct in 10 cases (17.5%), correct but incomplete in 9 cases (15.8%), and correct and complete in 37 cases (64.9%). ChatGPT o1 responses were graded as incorrect in 0 cases, partially correct in 9 cases (15.8%), correct but incomplete in 3 cases (5.3%), and correct and complete in 45 cases (78.9%) (Table 1). Differences between the two models were not significant (U = 1468; *p* = 0.1).

**Figure 2:**
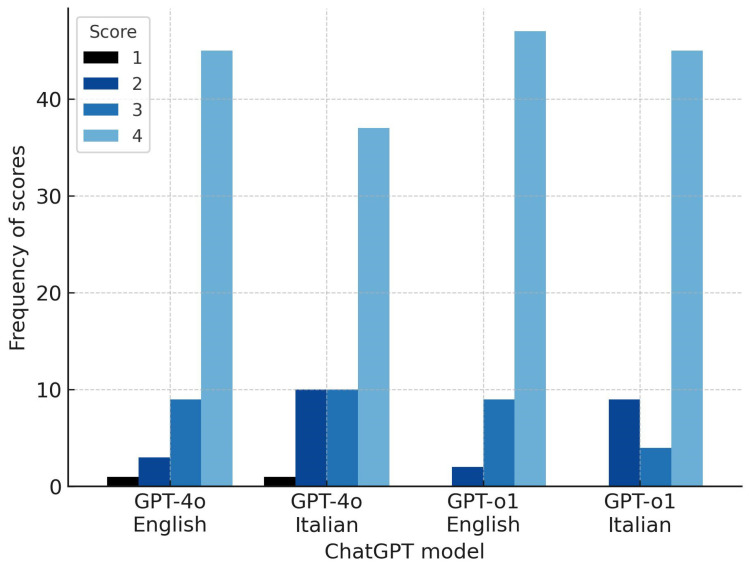
Grouped bar chart displaying the distribution of scores (from 1 = incorrect to 4 = correct and complete) assigned to responses provided by ChatGPT 4o and ChatGPT o1, separately for questions posed in English and Italian.

Responses in English generally received higher scores than those in Italian, particularly for ChatGPT 4o, although the differences were not statistically significant (ChatGPT 4o: U = 1374; *p* = 0.07; ChatGPT o1: U = 1535; *p* = 0.5) (Figure 2). However, subgroup analysis by question domain (clinical data, instrumental diagnostics, and therapy) showed significantly better English responses by ChatGPT 4o in clinical data questions (U = 302; *p* = 0.02), with no significant differences for other subgroups or ChatGPT o1.

**Figure 3:**
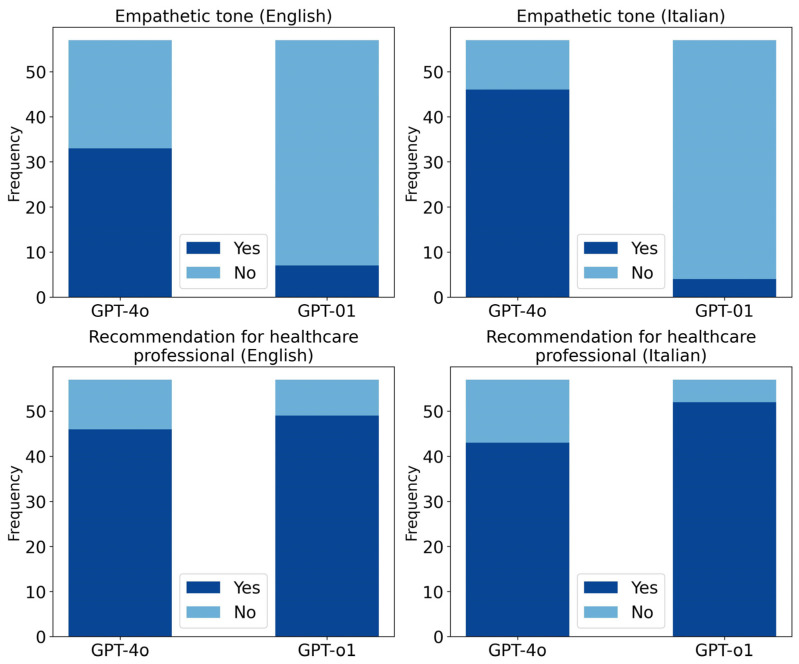
Stacked bar charts comparing responses of ChatGPT 4o and ChatGPT o1. The upper panels show the frequency of responses rated as empathetic in English (left) and Italian (right). The lower panels display the frequency of responses including a recommendation to consult a healthcare professional, again separated by language (English, left; Italian, right).

ChatGPT 4o responses were significantly more empathetic than those of ChatGPT o1 in both English and Italian (English: χ^2^ = 24.1; *p* < 0.0001; Italian: χ^2^ = 59.9; *p* < 0.0001) (Figure 3). ChatGPT o1, however, recommended consulting a healthcare professional significantly more often than ChatGPT 4o in Italian responses only (χ^2^ = 4; *p* = 0.04) (Figure 3).

## 4. Discussion

The main finding of this study was that both ChatGPT models provided a notably high proportion of responses to questions about prolonged DoC that were graded as either correct and complete or correct but incomplete, ranging from 80.7% for ChatGPT 4o in Italian to 96.8% for ChatGPT o1 in English. When comparing responses in English versus Italian, ChatGPT 4o—but not ChatGPT o1—performed significantly better on clinical data questions when these were posed in English. Additionally, ChatGPT 4o’s responses were rated as more empathetic than those of ChatGPT o1 in both languages. Conversely, ChatGPT o1 was more likely to recommend consulting a healthcare professional, although this difference was only significant in the Italian responses.

The reliability of medical information provided by large language model-powered chatbots is steadily improving, and, in some cases, has even been deemed superior to that of clinical experts in terms of accuracy, completeness, and empathy [27,28,29,30]. Accordingly, it is unsurprising that most responses in this study were rated as correct across both models and languages. Both models generally produced extensive answers, averaging approximately 320 words per response for ChatGPT 4o and 450 words per response for ChatGPT o1. As a result, most correct responses were also graded as complete, while only a relatively small percentage (5.3–15.8%) were considered correct but incomplete. However, the responses often included information beyond the specific scope of the questions. This verbosity may stem from several factors, including the training process, which tend to reward detailed and comprehensive answers. Notably, such verbosity has already been documented for ChatGPT in both medical and non-medical contexts [31,32,33].

Partially correct responses with significant errors accounted for 3.5% to 17.5% of cases, depending on the ChatGPT model and language. These responses contained both accurate and inaccurate elements. Additionally, two responses were rated as incorrect and potentially misleading: both concerned instrumental diagnostics and were generated by ChatGPT 4o. In one case, an English-language question asked whether somatosensory evoked potentials (SSEPs) could determine if a patient with a prolonged DoC was conscious. ChatGPT 4o stated that SSEPs can help distinguish between UWS and MCS; however, SSEPs are primarily used for prognostic purposes, and the response failed to mention the importance of considering the patient’s etiology when interpreting the results [34,35]. In the second case, an Italian question asked whether an electroencephalogram (EEG) could help determine if a patient is conscious. Multiple errors were identified in the response, including the incorrect claim that EEG can detect brain activity indicating whether the brain is minimally responsive or completely unresponsive, as well as the misattribution of the burst-suppression pattern to patients in UWS and MCS. Recognizing and understanding these error patterns could inform the development and refinement of future chatbot models, potentially enhancing their accuracy and clinical reliability.

An interesting finding of this study concerns the accuracy of responses provided in Italian compared to the reference language, English. While both ChatGPT models demonstrated strong overall performance in both languages, English responses generally achieved higher accuracy scores, particularly with the ChatGPT 4o model. Notably, this difference reached statistical significance within the clinical data subgroup, suggesting that language may influence the precision of information delivered by large language models on specific medical topics. These findings align with the well-documented predominance of English in the training datasets used to develop ChatGPT models, highlighting a potential linguistic bias in non-English contexts. Given the widespread use of such chatbots by non-English-speaking populations, these results underscore the importance of further refining multilingual training processes to ensure consistently accurate and reliable medical information across languages.

Caregivers of patients with DoC frequently experience significant burdens, including symptoms of depression and anxiety [36,37], underscoring the importance of empathetic communication when addressing concerns about a relative with a prolonged DoC. A key finding of this study was that ChatGPT 4o provided significantly more empathetic responses than ChatGPT o1, in both English and Italian. Notably, all prompts were framed as if posed by a patient’s relative; in this context, ChatGPT 4o’s greater empathetic tone may enhance its role as a supportive tool for families. Finally, both models frequently recommended consulting a healthcare professional, although in Italian, this suggestion was made more often by ChatGPT o1.

This study has some limitations. First, the questions used were derived from the authors’ extensive clinical and research experience with prolonged DoC. However, involving actual caregivers or family members in formulating the questions would have ensured greater alignment with the specific informational needs of relatives of patients with prolonged DoC. Additionally, consistent with previous studies evaluating chatbot responses to medical questions, we employed a four-point grading scale to assess the responses provided by the two ChatGPT models. While interrater agreement between the two primary reviewers was high, we acknowledge that reviewers with different professional backgrounds or expertise in prolonged DoC might interpret the grading criteria differently, potentially affecting the assigned scores. Finally, empathy was assessed by verifying the presence of explicitly empathetic statements, rather than using a more structured grading system, which may limit the standardization and comparability of our findings.

## 5. Conclusions

Both ChatGPT models demonstrated high accuracy in providing information on prolonged DoC, underscoring their potential as supportive tools for caregivers. In particular, the more empathetic responses generated by ChatGPT 4o may further enhance its value, particularly in emotionally sensitive contexts. Integrating chatbots into long-term care and rehabilitation pathways for individuals with prolonged DoC entails both significant opportunities and notable challenges. These tools could serve as readily accessible resources for caregivers, providing not only timely, accurate information, but also emotional support in situations often characterized by profound uncertainty and distress. Additionally, they could be used as educational tools, helping caregivers better understand the patient’s condition and navigate complex, long-term decisions. From a healthcare perspective, chatbots can also facilitate more effective communication between professionals and patients’ families, assisting in the management of delicate and emotionally charged interactions. Nonetheless, the occasional inaccuracies observed in this study highlight the need for careful implementation and continuous professional oversight. Future research should further explore the real-world impact of these tools on caregiver burden, patient outcomes, and their long-term feasibility within healthcare settings.

## Figures and Tables

**Table 1:** Grading of responses provided by two ChatGPT models (ChatGPT 4o and ChatGPT o1) to questions concerning prolonged DoC. Results are presented separately for three question domains (clinical data, instrumental diagnostics, and therapy), each posed in English and Italian. Data are reported as absolute values with corresponding percentages in parentheses.

Category	Questions in English		Questions in Italian	
	ChatGPT 4o	ChatGPT o1	ChatGPT 4o	ChatGPT o1
**Clinical data (*n* = 29)**				
Incorrect and potentially misleading	0 (0%)	0 (0%)	0 (0%)	0 (0%)
2. Partially correct with significant errors	1 (3.4%)	2 (6.9%)	5 (17.2%)	6 (20.7%)
3. Correct but incomplete	2 (6.9%)	5 (17.2%)	6 (20.7%)	2 (6.9%)
4. Correct and complete	26 (89.7)	22 (75.9%)	18 (62.1%)	21 (72.4%)
**Instrumental diagnostics (*n* = 14)**				
Incorrect and potentially misleading	1 (7.1%)	0 (0%)	1 (7.1%)	0 (0%)
2. Partially correct with significant errors	0 (0%)	0 (0%)	3 (21.4%)	3 (21.4%)
3. Correct but incomplete	2 (14.3%)	2 (14.3%)	1 (7.1%)	0 (0%)
4. Correct and complete	11 (78.6%)	12 (85.7%)	9 (64.3%)	11 (78.6%)
**Therapy (*n* = 14)**				
Incorrect and potentially misleading	0 (0%)	0 (0%)	0 (0%)	0 (0%)
2. Partially correct with significant errors	2 (14.3%)	0 (0%)	2 (14.3%)	0 (0%)
3. Correct but incomplete	4 (28.6)	1 (7.1%)	2 (14.3%)	1 (7.1%)
4. Correct and complete	8 (57.1)	13 (92.9%)	10 (71.4%)	13 (92.9%)

## Data Availability

The data supporting the findings of this study are provided in the article and its Supplementary Materials. Further inquiries can be directed to the corresponding author.

## Supplementary Materials

The following supporting information can be downloaded at: https://www.mdpi.com/article/10.3390/brainsci15040392/s1: ChatGPT Responses (English and Italian) and reviewers’ scores.

## Abbreviations

The following abbreviations are used in this manuscript:

DoC	Disorders of consciousness
UWS	Unresponsive wakefulness syndrome
MCS	Minimally conscious state
SSEPs	Somatosensory evoked potentials
EEG	Electroencephalogram
